# Essen transition model for neuromuscular diseases

**DOI:** 10.1186/s42466-022-00206-8

**Published:** 2022-09-05

**Authors:** Michael Fleischer, Bayram Coskun, Benjamin Stolte, Adela Della-Marina, Heike Kölbel, Hildegard Lax, Michael Nonnemacher, Christoph Kleinschnitz, Ulrike Schara-Schmidt, Tim Hagenacker

**Affiliations:** 1 Department of Neurology and Center for Translational Neuro- and Behavioral Science, University Medicine Essen, Hufelandstraße 55, 45147 Essen, Germany; 2 Division of Neuropediatrics, Department of Pediatrics 1, University Medicine Essen, Essen, Germany; 3 Institute of Medical Informatics, Biometrics, and Epidemiology, University Hospital Essen, Essen, Germany; 4 Center for Clinical Trials, University Medicine Essen, Essen, Germany

**Keywords:** Transition, Neuromuscular diseases, Late-onset Pompe disease, Juvenile myasthenia gravis, Duchenne muscular dystrophy, The concept of care, Interdisciplinarity

## Abstract

**Background:**

With the optimization of medical care structures and the rapid progress in the development of new therapeutic methods, an increase in life expectancy is observed in patients with neuromuscular diseases. This leads to an expansion of the phenotypic spectrum, whereby new or previously less relevant disease manifestations in different organ systems gain more importance. The care of adolescents and young adults with neuromuscular diseases, therefore, requires increasingly close interdisciplinary collaboration within neuromuscular centers.

**Research question:**

How can the transition process from pediatric to adult care be structured so that the individual disciplines are efficiently integrated into the complex treatment and care process, and the patients' quality of life is improved?

**Material and methods:**

A structured transition process was established at the University Hospital in Essen, Germany. Exemplarily, a comparable care concept was developed based on Pompe disease, Duchenne muscular dystrophy, and juvenile myasthenia gravis comprising four elements: (1) With the introduction of cross-department standard operating procedures, the logistical processes, as well as the diagnostic and therapeutic measures, are uniformly coordinated, and the transition process is bindingly defined. (2) To ensure a seamless transition, young patients are seen with their parents during joint consultations before they reach their 17th birthday. This creates an opportunity for patients to get to know the subsequent department structure and build a lasting relationship of trust. (3) A quarterly “transition board” regularly brings together the participating disciplines from pediatric and adult care systems for a case-related interdisciplinary exchange and continuous optimization of the transition process. (4) A cross-department “Transition Database”, in which medical findings and parameters are recorded, was implemented as a common information platform and database.

**Conclusion:**

The Essen Transition Model aims to close the gap in care for young patients with neuromuscular diseases during the critical transition from pediatric to adult medicine and to create a successful continuation of treatment in adulthood.

## Background

Almost 40% of children and adolescents in Germany suffer from a chronic disease, with about 14% having special health care needs [1]. Due to a steady improvement of therapeutic options and the expansion of care structures in the field of neuropediatrics, more and more adolescents with severe neuromuscular diseases reach adulthood [2]. Neuromuscular diseases are more and more to be understood as multi-system diseases. Due to increasing life expectancy, an expansion of phenotypic disease spectra with new or previously subtle organ manifestations is observed [3]. This necessitates more in-depth interdisciplinary treatment that extends beyond the period of pediatric care. To enable the smoothest possible transition from adolescence to adulthood, special attention should be paid to the often-complex disease-specific medical situation in addition to the “general” psychosocial and personal development [4]. Since patients, including their parents, have often been cared for in fixed care structures for many years until they reach adulthood—in some cases from birth onwards—an appropriate transition into the adult medical system with as little loss of information as possible is a particular challenge for the healthcare system and the adolescent patients.

The term “transition” describes the transfer of young people with chronic diseases from pediatric and adolescent medicine to adult medicine. The need for a structured transition process has been given increasing importance in health policy, both nationally and internationally [1, 5–7]. In Germany, a multidisciplinary guideline was also established with the foundation of a society for transition medicine [8]. The first systematic programs, such as the Berlin Transition Program, already exist, but nationwide coverage is still far off [10]. Inadequate transition can have far-reaching consequences for the health of adolescents. For example, without continuous and high-quality interdisciplinary care during the transition to adult medicine, serious complications such as transplant rejection in organ transplanted patients may occur [11]. Another example may be the transfer of adolescent patients with type 1 diabetes mellitus; here 40% of patients lose contact with qualified diabetology, which has been shown to result in an increasing risk of hyperglycemia [9]. Compared with adolescents who remained in pediatric care, adolescents had a 2.5-fold increased risk of adverse glycemic control, including associated sequelae, after transfer to adult medicine. Conversely, structured transition programs with individual case management were able to reduce the rate of sequelae from 40% to about 10% [12].

In everyday clinical practice, transition often occurs as an unplanned process as soon as further treatment in the pediatric field is no longer possible. This usually occurs on a “cut-off date”, usually when the patient reaches the age of formal adulthood, or in the worst case even in the course of a medical emergency [13]. This often leads to an unstructured and incomplete handover and does not cover the complex medical and social situation of those affected. This also jeopardizes the success of previous therapeutic efforts over many years, which can sometimes have far-reaching medical and social consequences [14, 15]. About 30–40% of affected adolescents confirm problems regarding transition [1]. However, a failed transition may not only have negative health consequences for the individual but is also highly relevant in terms of health policy and society. Targeted support into adult medicine can reduce the risk of complications, hospitalization, sequelae, early retirement, and ultimately costs to the health and pension systems [16]. Structured transition programs should therefore be seen as an opportunity to ensure improved treatment that also conserves resources [17].

In summary, a successful transition is characterized by a structured and coordinated transfer of adolescent patients to adult medicine. This requires the establishment of continuous, interdisciplinary and patient-oriented care structures of high quality. Currently, however, such a structured transition rarely takes place. For the transition of patients with neuromuscular diseases, the “Essen Transition Model” (ETM) was established, which is presented in the following. The “ETM” is aimed at all centers concerned with the transition of young patients to medical care in adulthood and meant to be a blueprint for establishing the process. Either the whole package or single parts could be adopted, depending on the circumstances in the clinical environment.

## Challenges of transition

The main challenge of a successful transition is to coordinate numerous individual elements and to interlock them in such a way that an efficient overall process that is feasible in everyday clinical practice and yet of high quality is achieved. In a 2009 report by the "Sachverständigenrat zur Begutachtung der Entwicklung im Gesundheitswesen", two major problem areas of transition were identified. On the one hand, difficulties were presented at the system level in the form of financing and organizational deficits, and on the other hand, deficits at the professional and social levels.

One major system factor impeding transition is inadequate reimbursement for the increased treatment demands of adolescents in special care situations. For example, patients in transition are treated by at least two specialists in each discipline at the same time. Other systemic barriers include a lack of qualified staff, a lack of training for caregivers, and a lack of uniform regulations on the optimal time to initiate the transition process (Sachverständigenrat zur Begutachtung der Entwicklung im Gesundheitswesen 2009). Furthermore, a comprehensive medical history of the young patients is often missing and documentation is fragmented, leading to unnecessary diagnostics or shortage of medical care. In view of these facts, comprehensive, specific education and training of medical personnel is of particular importance [18–20]. Further training in the field of transition for medical specialists has already been regulated in other countries, albeit only in rudimentary form to date. In the United Kingdom, for example, a curriculum ("Adolescent Health Program") has been developed that is accessible as an online program for medical specialists [21].

On a professional level, practitioners from adult medicine often feel inadequately prepared for the care of chronically ill adolescents [22, 23]. Compared to pediatric and adolescent medicine, the treatment of adults is often more disease-oriented and less "holistic" person-oriented or individualized [24]. This can be an unfamiliar situation for the patient, which can lead to uncertainty. Another important aspect is the patient's parents, whose care, although well-intentioned, may interfere with the personal development and independence of young adults, even in the medical field [25]. Also, the relationship between caregivers and previous treatment providers can be an obstacle in that “separation” can be difficult at this level as well in the case of a particularly intimate relationship [26]. This is a new challenge for practitioners from adult medicine, who are confronted with three (patient and parent) contacts during transition.

## The Essen model—for a successful transition

A structured transition process for patients with neuromuscular diseases has been established at Essen University Medical Center, which has been evaluated using the examples of late onset Pompe disease (LOPD), Duchenne muscular dystrophy (DMD) and juvenile myasthenia gravis (jMG).

The “Essen Model” is composed of the following elements:

- 1. Inter-disciplinary standard operating procedures (SOPs).
- 2. Transition consultation.
- 3. Interdisciplinary case conference ("transition board").
- 4. Transition database.

## Inter-disciplinary SOPs

Inter-disciplinary SOPs were created by the practitioners from the respective departments who are directly involved in the treatment process. This standardized the performance of diagnostic steps as well as the indication and performance of specific therapies. For example, the SOPs are intended to ensure that patients with a confirmed diagnosis of LOPD undergoing enzyme replacement therapy (EET) are subjected to standardized examinations with uniform recording of findings across all clinics. The process of SOP creation was accompanied by the hospital's own quality management, which makes the SOPs available to all participants in a document management system.

## Transition consultation

Identification and admission for or into the transition process take place when the adolescent reaches the age of 16. In a transition consultation, the adolescents and their relatives are examined by a treatment team consisting of one medical representative each from neuropediatrics and neurology, and the previous course and treatment are discussed, as well as the further diagnostic and therapeutic procedure. Two joint examination appointments within 12 months provide the opportunity for patients and relatives to get to know the continuing care provider as a future interaction partner and to identify possible difficulties in the transition from pediatric and adolescent medicine to adult neurology in a timely manner. Within the framework of the transition consultation, in addition to the steps necessary for the continuation of diagnostics and therapy, any future necessary co-care by other specialist disciplines is planned and coordinated (e.g., pneumology, cardiology).

**Fig. 1:**
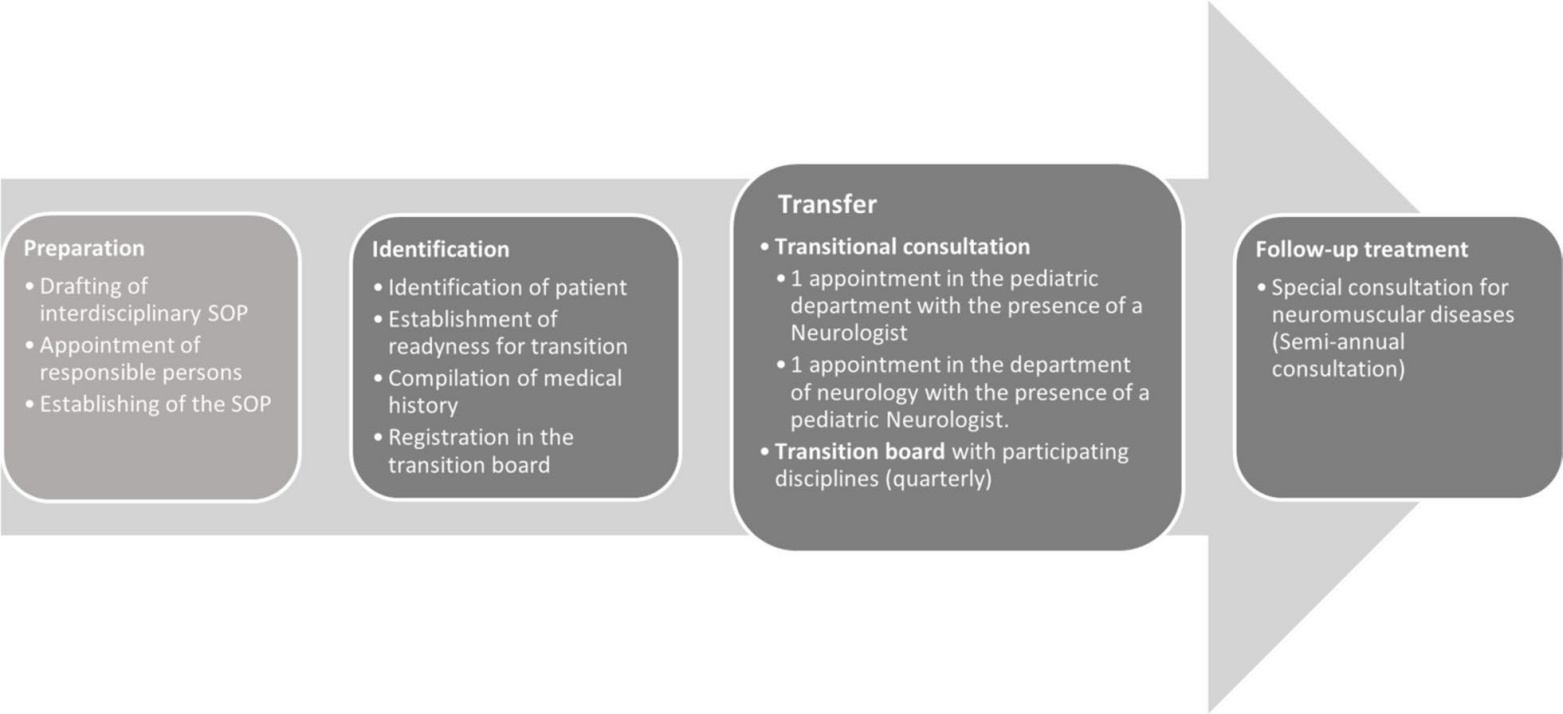
Ilustration of the transition process for neuromuscular diseases within the framework of the "Essen Transition Model". In the course of the preparations, the process was coordinated across clinics and bindingly defined. The process is divided into three sections ("Identification", "Transfer" and "Follow-up treatment"), within each of which specific tasks were defined

Upon reaching the age of 18, further care will then be provided by the adult neuromuscular outpatient clinic and patients will continue to receive care according to the treatment standards there (Fig. 1).

## Interdisciplinary case conference (“transition board”)

In other disciplines such as oncology, interdisciplinary case conferences (tumor boards) are already firmly established as an instrument for managing individual treatment. Analogously, a case conference in the field of neuromuscular diseases can facilitate the coordination of interdisciplinary diagnostic and therapeutic measures. Accordingly, a transition board has been established, which takes place quarterly and includes the respective specialist disciplines from the pediatric and adult areas. This supports an efficient workflow within the transition. The results of the transition board are documented on a case-by-case basis and stored in the transition database.

## Transition database

The basis of a successful transition is the effective exchange of information over an often long course of disease, across different treatment disciplines. This requires a common platform that makes treatment and progression parameters available to all therapists across clinics. In addition to the basic data of the patients, diagnostic and functional parameters during the course of the disease, further therapy-relevant information, information on the provision of medical aids and remedies, as well as socio-medical information are made available. In order to maintain a disease-specific focus and to avoid creating a parallel structure to the existing hospital information system, the necessary parameters and data were agreed upon in a consensus-building process with the disciplines involved. With this, the database aims to assist in creating a comprehensive medical history that could be provided to all disciplines included in the treatment and forms the basis for the transitional consultation.

**Fig. 2:**
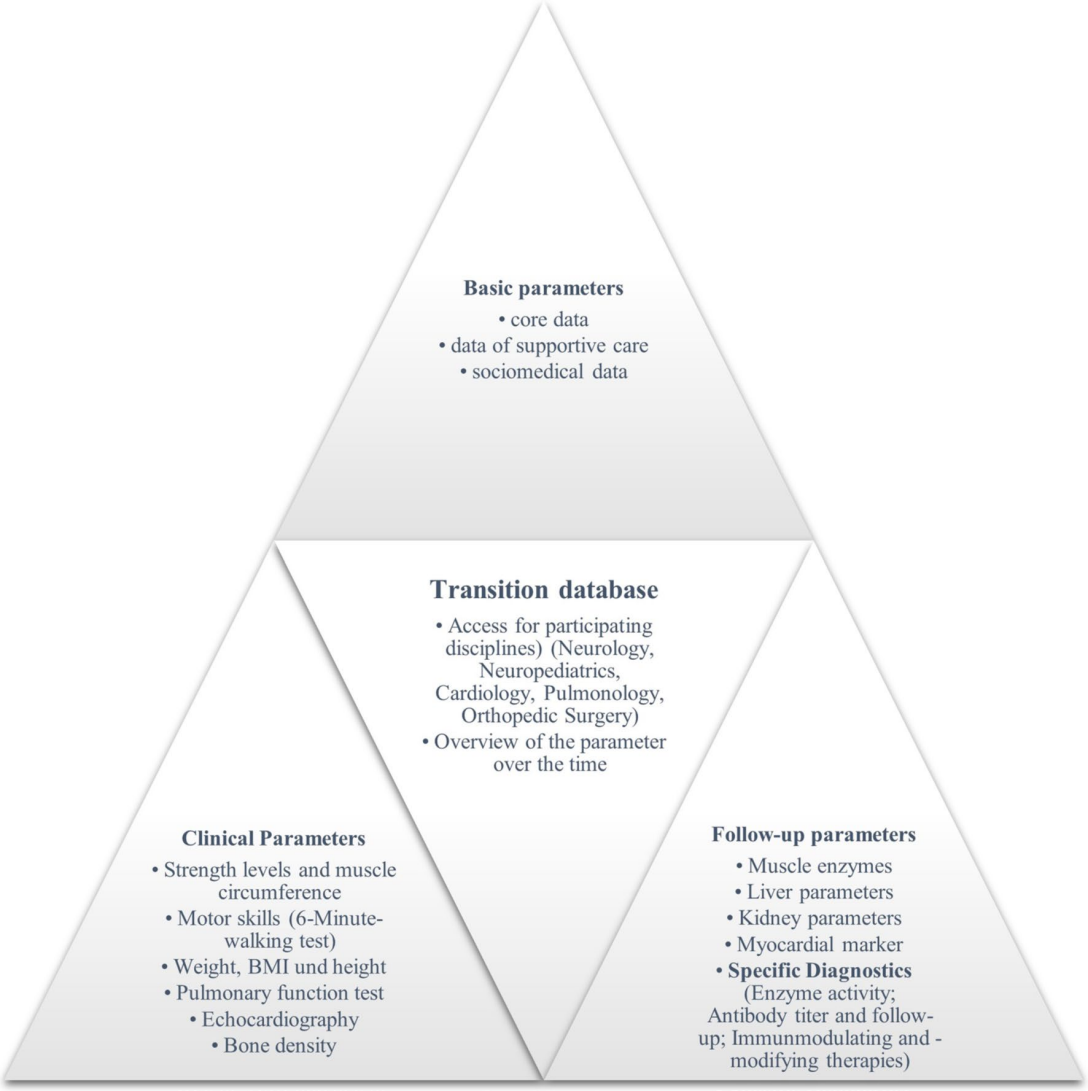
Representation of the parameters that are included in the transition database and are made available across disciplines and clinics as well as over time. The parameters are divided into the groups basic parameters, clinical parameters and follow-up parameters

The parameters specifically collected for neuromuscular diseases include, for example, height, weight, BMI, abdominal circumference, laboratory parameters (CK, CK-MB, liver enzymes, kidney values, antibody titers against alfaglucosidase in LOPD) and human genetic findings. To assess the progression of musculoskeletal symptoms, upper and lower extremity circumferences and motor assessment and progression scores (e.g., motor milestones, Brooke Upper Extremity Scale for upper extremity function, 6-min walking test to assess walking ability and muscular endurance) are recorded in addition to individual strength grades. Pulmonary and cardiac performance is mapped by measuring vital capacity (VC), forced one-second capacity (FEV1), ejection fraction (EF), and fractional shortening (FS) (Fig. 2).

A 6-month presentation interval provides a comprehensive status picture that allows longitudinal assessment of disease progression and often response to therapy. Conversely, the need for intervention or further therapeutic measures is identified at an early stage. The recording of information on the provision of aids and remedies as well as the recording of socio-medical aspects are intended to round off the overall picture of the individual patient and prevent under- or overprovision.

Maintenance of the database and preparation of the transition consultation and interdisciplinary transition board is transferred to a “case-manager”, supporting and reducing workload of the healthcare professionals. With the embedding of the database in the documentary system of the hospital not only data security and privacy are guaranteed but also that the collection of data is fully conform with GCP requirements.

## Outlook: scientific analysis of the transition process

**Fig. 3:**
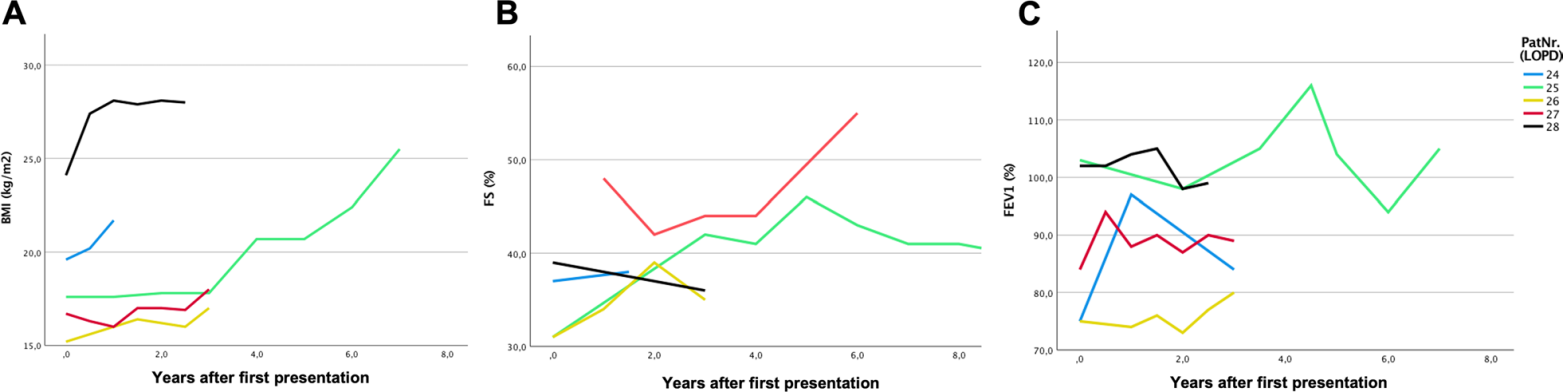
Exemplary representation of clinical course parameters over time for individual patients with LOPD on enzyme replacement therapy. Shown are the courses from the time of presentation. **A**) BMI shows predominantly an increase over time. **B**) Fractional shortening (FS) over time. **C**) Change in one-second capacity (FEV1) over time

The creation of the transition database creates an information platform for all treatment partners involved within the university medical center by enabling the recording of parameters and course parameters. By means of a visually clear mask and pre-entries, the effort for documentation is kept low. In the pilot phase, existing data for the selected model disease patterns (LOPD, DMD, and jMG) were collected retrospectively. In addition to demographic information (Table 1), an exemplary query of some clinical course parameters (lung function, fractional shortening, and BMI) (Fig. 3) is shown. In addition to the progression parameters, the development of a transition score is planned. In this, the pediatric progression parameters and assessment scores will be translated into adult medicine. This could establish a parameter for the evaluation of transition quality. Disease progression could be better documented over time and the necessity of, for example, a change in therapy during adolescence could be more easily recognized.

**Table 1:** Cohort of patients within the analysed transition care phase

	LOPD	DMD	jMG
Number	10	20	5
*Sex*			
Male	6	20	–
Female	4	–	5
Age	30,1	24,9	22,2
BMI	22,0	23,6	21,5

## Funding of transitional process

The counter financing of transition services continues to be a problem. The current status is still that of a project, nonetheless, the process should become part of regular care, which would require adequate funding. Although, neurologic patients make up a relevant proportion of all patients needed to be translated across all specialties, a coordinative position of a “Care manager” will not be fully realized. We are therefore trying to establish a joint “Center for Translation”, which coordinates translational processes and maintained, across the various disciplines. Furthermore, with the implementation of such concepts as the ETM, the basis for a structured data collection is created, which generates real-world data about the care situation and the translation process. This data can then be used as a basis for negotiating reimbursement with the health insurance companies via a corresponding billing code.

## Conclusion for practice

- Better care and new therapeutic options are extending life expectancy in patients with neuromuscular diseases and new phenotypes are emerging that have not been observed previously.
- A close exchange between the treating disciplines is important due to the complex neuromuscular disease patterns.
- A structured transition process improves treatment adherence and the quality of life of young patients.
- The “Essen Transition Model” includes individual components that can serve as a template for other centers to establish a structured transition process with adaptation to local infrastructural conditions.
- For the future, recommendations from the German S3 guideline on transition should also be usefully incorporated into the process.
